# Patterns of direct observation and their impact during residency: general practice supervisors’ views

**DOI:** 10.1111/medu.13631

**Published:** 2018-07-24

**Authors:** Chris B T Rietmeijer, Daniëlle Huisman, Annette H Blankenstein, Henk de Vries, Fedde Scheele, Anneke W M Kramer, Pim W Teunissen

**Affiliations:** ^1^ Department of General Practice and Elderly Care Medicine VU University Medical Centre Amsterdam The Netherlands; ^2^ School of Medical Sciences VU University Medical Centre Amsterdam The Netherlands; ^3^ Athena Institute for Transdisciplinary Research VU University Amsterdam The Netherlands; ^4^ Department of Public Health and Primary Care Leiden University Medical Centre Leiden The Netherlands; ^5^ School of Health Professions Education Maastricht University Maastricht The Netherlands

## Abstract

**Context:**

Direct observation (DO) of residents’ performance, despite the importance that is ascribed to it, does not readily fit in with the practice of postgraduate medical education (PGME); it is infrequent and the quality of observation may be poor in spite of ongoing efforts towards improvement. In recent literature, DO is mostly portrayed as a means to gather information on the performance of residents for purposes of feedback and assessment. The role of DO in PGME is likely to be more complex and poorly understood in the era of outcome‐based education. By exploring the possible complexity of DO in workplace learning, our research aims to contribute to a better use of DO in the practice of PGME.

**Methods:**

Constructivist grounded theory informed our data collection and analysis. Data collection involved focus group sessions with supervisors in Dutch general practice who were invited to discuss the manifestations, meanings and effects of DO of technical skills. Theoretical sufficiency was achieved after four focus groups, with a total of 28 participants being included.

**Results:**

We found four patterns of DO of technical skills: initial planned DO sessions; resident‐initiated ad hoc DO; supervisor‐initiated ad hoc DO, and continued planned DO sessions. Different patterns of DO related to varying meanings, such as checking or trusting, and effects, such as learning a new skill or experiencing emotional discomfort, all of them concerning the training relationship, patient safety or residents’ learning.

**Conclusions:**

Direct observation, to supervisors, means much more than gathering information for purposes of feedback and assessment. Planned DO sessions are an important routine during the initiation phase of a training relationship. Continued planned bidirectional DO sessions, although infrequently practised, potentially combine most benefits with least side‐effects of DO. Ad hoc DO, although much relied upon, is often hampered by internal tensions in supervisors, residents or both.

## Abstract 2

The authors offer explanations for the lack of direct observation (DO) in residency by exploring its manifestations, meanings and effects in training relationships; DO may benefit from pre‐planning and bi‐directionality.

## Introduction

Direct observation (DO) of residents’ performance, despite the importance that is ascribed to it for purposes of feedback and assessment,^1^, ^2^, ^3^, ^4^, ^5^ does not readily fit in with the practice of postgraduate medical education (PGME); it is infrequent and the quality may be poor in spite of ongoing efforts to improve this.^4^, ^6^, ^7^, ^8^, ^9^, ^10^, ^11^ By DO, we mean that the supervisor is physically present, watching the resident providing patient care. In the literature on PGME, DO is predominantly seen as an important means of gathering information on the performance of the resident, for purposes of supervision, feedback, assessment and entrustment.^5^, ^7^, ^10^, ^12^, ^13^ Recently published guidelines on DO in medical education confirm this instrumental approach to DO as a ‘key assessment strategy in competency‐based medical education’.^11^ Given the evident and broadly advertised importance of this information‐gathering aspect of DO, there must be other aspects that explain the infrequent use of DO in the practice of PGME. Recent literature has uncovered some of these: residents report that anxiety, caused by fear of assessment, affects their performance when observed directly.^8^, ^14^ In addition, cultural values in the workplace, such as efficiency and residents’ autonomy, may conflict with residents asking for, and supervisors offering, DO.^15^, ^16^, ^17^

Another aspect of DO in training relationships, relatively underexposed in the literature on DO, is that it may work in two directions. Historically, DO was an important early step in workplace learning processes. In the traditional master–apprentice relationship, apprentices would observe their masters performing specific tasks.^18^ The apprentices would then mimic these performances, gradually taking over the tasks while being observed by their masters. Observation was thus an interplay of mimicking by the apprentice and guiding and assessing by the master, while together working on a task; in other words, observation worked two ways, both resident and supervisor observed and were observed. By contrast, current conceptualisations of DO seem to approach DO as a one‐way process, informing feedback and assessment of the resident.^11^

When trying to understand the lack of DO for feedback and assessment, a broader look at DO in workplace learning could be insightful. We know from the literature that DO is one possible level of supervision, to be distinguished from nearby, immediately available supervision, and from more distant supervision.^19^ Supervisors need to make judgements as to which level is appropriate with regard to patient safety and residents’ developmental trajectories.^19^, ^20^ The development of trust plays a role in this process^12^, ^20^ and the length and quality of the relationship between supervisor and resident are further important factors.^11^, ^19^, ^20^ In short, DO in workplace learning within a developing training relationship between supervisor and resident may be a more complex phenomenon than has been recognised in recent literature on feedback and assessment. Understanding this complexity could help to make DO work better for residents’ learning. We therefore formulated the research question: What are the manifestations, meanings and effects of DO in developing postgraduate training relationships? We chose to focus on the supervisor's perspective because, according to the literature, supervisors play a pivotal role in the orchestration of DO in clinical practice.^11^, ^12^, ^19^, ^20^, ^21^ Understanding their perspective therefore seems to be essential. For this purpose, we conducted a constructivist grounded theory study using focus groups with supervisors of general practice (GP) residents.

## Methods

### Setting

We conducted our research in postgraduate GP training at the VU University Medical Center in Amsterdam, the Netherlands. Direct observation of communication skills is common in Dutch GP training.^9^, ^22^ By contrast, DO of technical skills is performed infrequently, reflecting literature on the lack of DO in other residency programmes.^9^, ^22^ We chose to focus on DO of technical skills only, for reasons of clarity, and to align with and add to the literature mentioned in the introduction.

In Dutch GP training, residents are paired with a GP supervisor, in whose GP they work for the first year of their training. The second year consists of internships outside GP. The third and final year is again in GP, this time with a new supervisor. Residents work under nearby supervision, with their supervisors immediately available in person. New GP residents may be recruited directly from medical school or after 1 or more years of experience in a hospital or another setting such as a nursing home. General practice supervisors have at least 5 years of experience as a general practitioner and participate in an ongoing faculty development course of 10 days per year, mostly in small groups of 10 to 15 persons, in which didactic issues relevant for GP supervisors are covered. Direct observation of technical skills was not specifically addressed during the period in which our focus groups were conducted.

### Study design

Constructivist grounded theory informed our data collection and analysis.^23^, ^24^ Our aim was not to test or verify existing theories, but to investigate what insights are gained when supervisors discuss DO of their residents’ technical skills in a broad sense. Discussing experiences in peer groups, with supervisors responding to each other and a facilitator probing to ensure deeper insights, seemed to be an appropriate research method to generate rich data; we therefore chose focus groups for data collection.

### Participants and procedure

We planned our focus groups with supervisors during regular faculty development courses, and as such our sampling method was convenience‐based. Purposively, we made sure we included groups of supervisors of first‐year as well as third‐year residents because we expected to find differences between the experiences of these groups. More senior residents tend to work more independently with less DO^16^, ^25^ and, with experience, supervisors differ in their approach to trust.^12^ In our institution, supervisors of third‐year residents are on average more experienced supervisors. The focus group sessions were conducted between June and December 2016, with each one lasting approximately 75 minutes. Focus groups were held until theoretical sufficiency was reached. Supervisors received information on our research and an invitation to participate in a focus group; they were then free to accept or decline without any repercussions. All participants gave written informed consent. The principal researcher (CBTR) was present at all focus group sessions and moderated the discussion in three of the four focus groups. In our efforts to be as open‐minded as possible, we had one focus group moderated by a moderator (AdW) who was not part of our research group and not associated with our training institute. All discussions were audiorecorded; DH was present at all meetings and had a passive observational role. She made notes that could be of value in the later stage of interpreting the data during the analysis. CBTR, researcher and senior staff member of the GP training institute, had no direct relationship with the participants. DH was not associated with the training institute.

Focus group discussions were summarised in 1300 to 1500 words and sent by e‐mail to participants, asking them to what extent the summary reflected what had been said and if they would like to add new ideas on the subject of DO. The purpose of this was to enrich our data.

### Interview guide

The initial interview guide was the result of a brainstorm with the authors CBTR, DH, AHB, HdV, FS and PWT. The guide was informed by the literature on DO as described in the introduction, and the authors’ experiences as clinicians and supervisors. After each focus group session, the interview guide was adapted, allowing for topics that had emerged to inform subsequent sessions. Because we were looking for all possible manifestations, meanings and effects of DO, we chose to start the focus group discussions with broad questions concerning DO of residents’ technical skills, and then to see what transpired, leaving ample room for participants to add matters that they felt related to DO. An example of a question is: When I ask you about direct observation of your residents’ technical skills, what comes to mind? All topics were likewise openly addressed to stimulate supervisors to contribute their thoughts and associations to the conversation, and the interview guide was used loosely. The topics addressed were: manifestations of DO of technical skills (How does DO occur? Who is present? Who does what? etc.); the supervisor's thoughts and feelings with regard to observing his or her resident; the assumed thoughts and feelings of residents with regard to being observed by a supervisor; the importance and benefits of DO; the initiative to engage in DO, and the influence of the relationship between supervisor and resident on DO and vice versa. An example of a topic that emerged during the first groups and was further highlighted in subsequent sessions was the importance of not observing directly.

### Analysis

A total of three supervisors chose not to participate for reasons of lack of time (n = 1) and lack of interest (n = 2); we included 28 supervisors (see Table 1). Group sizes ranged from four to 10 participants.

**Table 1:** Participant information for all four focus groups divided by year of residency

	*n*	Female/male	Age, years, mean (range)	Years of experience in GP, median (range)	Years of experience as a supervisor, median (range)
Supervisors of year 1 Focus groups 2 and 3	15	8/7	49 (39–62)	14.5 (7–30)	5 (0.5–22)
Supervisors of year 3 Focus groups 1 and 4	13	5/8	54 (46–66)	22.5 (10–35)	8 (4–25)

GP = general practice.

In response to the focus group summaries sent out to participants, we received some e‐mails with approval, but no additional suggestions. All audiorecordings were transcribed verbatim and entered into qualitative software (atlas.ti; Scientific Software Development GmbH, Berlin, Germany). Analysis was guided by the principles of constant comparative analysis as in constructivist grounded theory. After each focus group session CBTR and DH discussed emerging themes. Memo writing served to capture themes and evolving insights and questions about how themes may be connected. These insights and questions informed subsequent focus groups and guided the analysis process.

CBTR and DH independently coded and categorised quotes for the first three transcripts. They discussed all codes and categories until agreement was reached, and contributed to rigour by coding and discussing a random selection of quotes with NB, HdV and PWT. Then, by constantly comparing codes and categories over focus groups and consulting relevant literature, overarching themes were defined and a code book was developed. This code book was discussed with all the authors (except AWMK) until consensus was reached and refined by CBTR and DH. DH used the code book to code the remaining transcript; this was revised by CBTR, then discussed between CBTR and DH until agreement was reached.

After two focus groups, we constructed a first theoretical model that was refined through the third and fourth focus groups; in the fourth focus group no new insights came up to add to our theoretical model, indicating that theoretical sufficiency had been reached.^23^

The study protocol was approved by the ethics review committee of the Dutch Association for Medical Education (Nederlandse Vereniging voor Medisch Onderwijs [NVMO]).

## Results

An important finding in our early analysis was that supervisors strongly connected DO with not observing directly, but instead providing nearby, immediately available supervision. Discussing the meanings and effects of DO with supervisors therefore also implied discussing the meanings and effects of not observing directly. All participants indicated that in their practices, almost from the start, residents worked independently, seeing their own patients, in their own rooms under nearby supervision. Supervisors find this important because it gives the residents space to self‐regulate their learning, gain self‐confidence and develop their own working style:

You have to give them the confidence that they can solve problems by themselves […] If you nanny them too much by observing them directly, you make anxious GPs of them. (S3, FG3)

**Figure 1:**
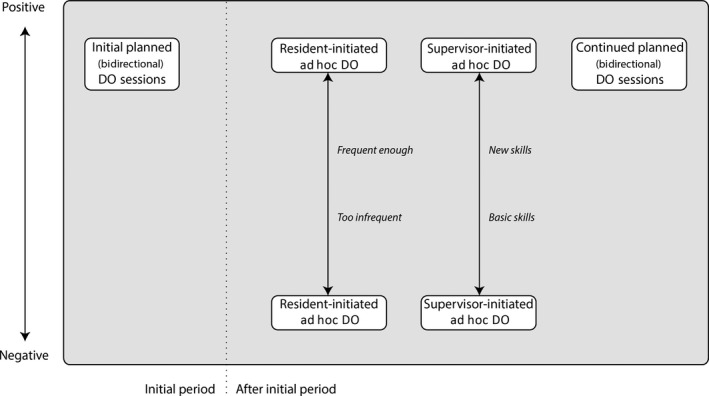
Patterns of direct observation (DO) and their reported impact on training relationships

Supervisors thus weighed DO against nearby supervision. A variety of perceived meanings and potential effects of DO played a role in decisions on when and how to engage in DO. Our data allowed us to identify four distinct patterns of DO; see Fig. 1 for a depiction of how these patterns impact training relationships according to our participants. We will describe these patterns with their associated meanings and effects in the following paragraphs.

### Pattern 1: initial planned (bidirectional) DO sessions

Most supervisors reported performing series of consultations together with their residents at the beginning of the training relationship. This was important because it enabled them to get acquainted and establish a working relationship and informed them about the resident's technical and communication skills, manner with patients and way of dealing with uncertainty and lack of experience:

Yes, first you have to watch for a while to … to get to know your resident and to gain confidence that he actually does have the abilities that he claims he has, that he isn't overestimating himself. (S7 FG2)

The overarching meanings of DO in these initial planned DO sessions, according to supervisors, are the securing of patient safety and residents’ learning. Concerning the latter, supervisors reported that, during these sessions, they teach by observing their residents and giving them feedback. They also teach by demonstrating skills, while the resident is observing them. Many supervisors reported taking turns with their residents in taking the lead in providing patient care, and thus alternating being the observer and being observed. This allows supervisors to combine checking their residents’ skills with teaching by showing and telling. We call this ‘bidirectional DO’:

Well, a new resident will first sit with me doing consultations, taking turns until I think, okay, now do it yourself. (S2 FG1)

Supervisors indicated that they find these bidirectional DO sessions very beneficial to residents’ learning of technical skills: how to apply them, when to apply them and how to interpret the results:

… and then they [the residents] say things like: ‘I've learned more about auscultation this week than in my entire medical training.’ (S4, FG2)

Additionally, supervisors reported that, during these sessions, they feel residents also learn from seeing them dealing with uncertainty and time pressure. The time and effort supervisors and residents spent on planned initial DO sessions varied. Some supervisors made a point of observing their resident performing most technical skills, whereas other supervisors tended to rely on a quick scan, for instance, arguing that it should not be necessary to check physical examination skills because residents are qualified doctors. In terms of invasive procedures, such as inserting an intrauterine device and minor surgery, most supervisors wanted to observe these at least once before they entrusted them to their resident.

#### Relational and emotional impact of DO

Besides the beneficial supervisory effects of initial planned DO sessions already mentioned, supervisors acknowledged the relational and emotional impact of DO of residents’ technical skills. Importantly, some DO patterns were more likely to cause emotional discomfort than others. We elaborate here on the general, omnipresent, although to varying degrees, relational and emotional impact of DO. These effects are also present alongside the effects of the other DO patterns discussed below.

Supervisors stated that their residents may feel uncomfortable during DO and that DO influences their performance. Supervisors themselves may also feel uncomfortable, for example when facing underperformance that has to be addressed. Also, some supervisors saw DO as a loss of time in their busy day. Many supervisors stated that DO is always difficult to start with, but that residents tend to get used to it. They described that over time supervisors and residents get to know each other and build mutual trust, especially if observation is performed routinely and bidirectionally:

I think a lot of residents find it very difficult, but they won't easily admit it. (S4, FG4)

I think taking turns during consultations, so that residents also observe their supervisor, is a good way of creating a safe atmosphere. (S2, FG3)

Relational aspects regarding the patient may cause some unease during DO sessions. Patients often turn to the supervisor when the resident is supposed to be leading the consultation. Supervisors reported several strategies to deal with these unclear and sometimes awkward communication patterns, such as:

I often have a sheet of paper in front of me so that I appear to be writing. Otherwise the patient, who is my patient after all, will indeed talk to me all the time [instead of to the resident]. (S2, FG2)

Explicitly clarifying the situation was seen as helpful, albeit seldom practised, in establishing good cooperation between all three parties.

As depicted in Fig. 1, supervisors positively value initial planned DO sessions, notwithstanding some possible discomfort.

### Pattern 2: resident‐initiated ad hoc DO

Asking the supervisor for help in patient care was very common, and during the focus groups supervisors indicated that they valued this as a sign of self‐regulated learning and that it meant that a resident was dealing professionally with uncertainties. Supervisors frequently came up with examples of these help‐seeking situations as a way of initiating DO. While exploring these examples, supervisors realised that they seldom ask the resident to demonstrate a technical skill in these situations:

… listening to the lungs … and then I don't look how she does it, but I listen to her, to how she interprets what she hears. I listen [to the lungs] too and then give my interpretation … (S2, FG4)

Thus, in these instances of help seeking, DO is mostly not of a technical skill but of clinical reasoning or of how the resident interacts with the patient. Sometimes, by contrast, residents ask for a specific observation of a technical skill. This mostly takes place as a planned teaching session.

Supervisors stated that they expected the resident to initiate a certain amount of ad hoc DO, although in the focus groups they were seldom explicit about the amount. As long as residents asked for enough ad hoc DO in the eyes of their supervisors, supervisors felt that patient safety and monitoring of the progress of residents’ learning were secured, while allowing for residents’ self‐regulated learning, autonomy and development of their own working style the rest of the time. Alongside mostly satisfactory effects of resident‐initiated DO, some possible emotional discomfort, as described under pattern 1, was still reported.

### Pattern 3: supervisor‐initiated ad hoc DO

Supervisors said they felt uncomfortable checking on their residents, fearing that the residents might feel they were being assessed or even mistrusted, and they reported that residents sometimes did express such feelings. The object of DO was pivotal here: supervisors felt that residents had little problem being observed while learning a new skill. By contrast, when DO was of basic skills that residents are expected to have learned in their undergraduate training, such as most physical examinations, supervisors were afraid that residents would feel mistrusted. Supervisors said they often feel uncertain or even bad when initiating ad hoc DO:

… if it is about skills of which they feel ‘actually I am supposed to have mastered this by now’, you know, like examining the shoulder or the abdomen or whatever … ‘and you come and watch whether I do it correctly’ then it feels like checking up on them or something … and that gives a different type of stress. (S7, FG4)

If residents ask for only a little DO, supervisors reported that they sometimes wonder if they are failing to ensure patient safety or falling short in their teaching. However, only a few supervisors reported that this leads them to initiate ad hoc DO, as long as there are no clear signs of residents underperforming. Moreover, most supervisors found it difficult, and sometimes even counterproductive, to impose DO on residents who did not ask for it or who avoided it:

Yes, I find that difficult, because … well … my last resident did not ask me very often, and then, […] if you want to observe more, you need to be a bit pushy. That's something I find difficult because it's like you're reversing the situation … if you first gave them responsibility, then it can feel like you no longer trust them … (S2, FG2)

Supervisors found it hard to discuss mutual expectations and needs openly in this situation. This pattern is where supervisors reported the most emotional discomfort, struggling to find a good balance between checking and trusting.

### Pattern 4: continued planned (bidirectional) DO sessions

After the first period with its self‐evident, planned series of mostly bidirectional DO (pattern 1), some supervisors continue to organise these DO sessions, for instance for 1 hour a week. They reported that these regular sessions mean that DO becomes a normal part of the training relationship, and that both supervisor and resident become accustomed to observing and being observed. Supervisors stated that these sessions often become a pleasant experience and give them a good impression of a resident's skills and progress. Supervisors acknowledge their own wishes for control and teaching, and at the same time they build a sound basis for trusting their resident to work without DO, allowing for autonomy and self‐regulated learning. In the focus group discussions, continued planned DO was often mentioned as a means of avoiding the difficulties that supervisor‐initiated ad hoc DO may potentially entail. Many supervisors, by contrast, said they stop continued planned DO sessions after the period of becoming acquainted, as described under pattern 1. Supervisors explained their refraining from continued planned DO sessions at this stage by referring to other occasions of working together during evening and night shifts. Also, they mentioned positive motives for not observing directly, like giving residents room for self‐regulated learning and the opportunity to develop their own working style and autonomy. Besides these positive motives, practical constraints such as time limitations also played a role, and many supervisors said they would like to organise these continued planned DO sessions but fail to do so:

Well, I have a very busy practice, so although I would like to work together for an hour and a half every week, seeing patients together, it just doesn't happen. Then it all becomes more haphazard … (S1, FG1)

Summarising the above patterns of DO, they appear to be defined by the stage of the developing relationship, who initiated DO, what was observed and how it was done, either ad hoc or as a planned session, either bi‐ or unidirectionally. Different patterns of DO related differently to a multiplicity of meanings and effects, like becoming acquainted, checking, teaching, trusting, mistrusting, giving space, loss of time, falling short, emotional discomfort and pleasure, all of which related to the training relationship, patient safety or residents’ learning. Clarity on the part of supervisors and residents regarding the intention with which DO was offered or requested appeared to be especially important in the ad hoc patterns of DO. Supervisors differed in their repertoires for dealing with situations where there was a clash between their role as teacher, residents’ independence and patient safety.

## Discussion

We set out to investigate the manifestations, meanings and effects of DO of technical skills in developing GP training relationships. We found that for our GP supervisors DO means much more than gathering information for purposes of feedback and assessment; indeed, the latter was hardly ever mentioned. In the following paragraphs we discuss how our findings resonate with the literature on the development of trust in supervisory relationships, underlining the role of DO in a solid start. Also, we will show how bidirectionality of DO is reflected in the literature on workplace learning. To the literature on DO for feedback and assessment we add the concept of DO patterns when thinking about initiation and organisation of DO.

### Direct observation at the beginning of the training relationship

Trust was a central theme when discussing DO in our focus groups. We know from the literature how mutual trust relates to the quality of the training relationship and how this quality may be affected by its duration.^19^, ^26^, ^27^ We add the importance of a solid start to the training relationship, and the role of DO in this. We found that in GP training relationships, typically prolonged, lasting 1 year, supervisors organised a solid start, using planned DO sessions for establishing initial trust. In a context similar to ours, Sagasser et al.^16^ found that supervisors reported similar needs to inform themselves early on about residents’ clinical competence and coping mechanisms. Amongst other strategies, they directly observed their residents to ensure patient safety and to gain insight into residents’ learning capabilities. Sheehan et al.^28^ added another insight as to why the initiation phase is important: in the context of interns’ clinical rotations, they found that this is the time when expectations are shared and preferences and idiosyncrasies of the workplace revealed. We found that supervisors naturally used planned DO sessions at the beginning of the training relationship as an opportunity to combine familiarising themselves with residents’ abilities with sharing expectations and preferences.

### Bidirectional DO

During initial planned DO sessions, supervisors not only observed their residents but made sure residents observed them too; this way they could teach them by demonstrating and discussing technical skills, and by demonstrating how they deal with uncertainty and time pressure. We have called this ‘bidirectional DO’. This finding aligns with the wider literature on vocational training. For instance, when interviewing workers from a range of occupations about how they learn through and for work, Billett found that much and perhaps most of our workplace learning results from working (i.e. being in the workplace, observing and listening and copying different behaviours).^29^, ^30^ The process of learning by observation and imitation is called mimesis.^29^, ^30^ Our participants recognised the power of this process and made sure that their residents would learn from observing their supervisors during planned bidirectional DO sessions. The bidirectionality of this process had further beneficial effects: it added to a safe atmosphere and a trusting relationship, while safeguarding patient safety and residents’ learning. Even though they recognised the potential of continued planned bidirectional DO sessions, many supervisors stopped organising these sessions after the initial period for reasons that were often unclear, although time constraints were mentioned.

### The difficulties of ad hoc DO patterns

Requesting ad hoc DO can be difficult for learners. Pelgrim et al.^14^ found in a setting similar to ours that most residents felt apprehensive about DO and some therefore avoided it. Research by Watling et al.^15^ revealed cultural obstacles; residents from a range of disciplines reported feeling responsible for initiating DO, while also wanting to meet culture‐based expectations concerning their autonomy and efficiency, thus becoming ‘conflicted learners’. We looked at the issue from the perspective of supervisors. In line with Watling et al.'s finding,^15^ supervisors did indeed hold their residents responsible for initiating ad hoc DO. However, by contrast, they did not report a conflict between their expectations concerning residents initiating ad hoc DO and their expectations concerning residents’ autonomy and efficiency, nor did they assume this played a role in the perception of the resident. This is an interesting discrepancy in findings that might indicate that supervisors are unaware of this particular aspect of residents’ struggle with initiating ad hoc DO. Another possible explanation for this discrepancy lies in the different cultural and educational contexts of both studies; our context of GP perhaps values help‐seeking behaviour more, or efficiency and autonomy less, than Watling et al.'s^15^ context of hospital disciplines. Adding to the issue of tension around initiation of ad hoc DO is the fact that supervisors reported that they struggled with residents who initiate very few instances of ad hoc DO, particularly when there are signs of underperformance. It leads supervisors to become conflicted: they feel they should initiate DO but they hesitate to do so, fearing that the resident might feel mistrusted. Together, these findings indicate that internal tension, both in residents and supervisors, may result in not initiating ad hoc DO.

### Implications for the practice of workplace learning

Our pattern framework, together with earlier research, may explain the poor fit of ad hoc DO in PGME: both supervisors and residents have their reasons not to initiate ad hoc DO. The emphasis on outcome and accountability in competency‐based medical education, operationalised as entrustable professional activities and programmatic assessment, has led to the development of observation tools and quantitative requirements for residents to assemble a number of DOs.^31^, ^32^ Our results help us understand why relying on ad hoc DO to meet these obligations has led to disappointing outcomes.^33^, ^34^, ^35^

In their guidelines on DO, Kogan et al.^11^ advocate sharing the responsibility for the occurrence of DO between the supervisor, the resident and the training programme, and making DO sessions routine. Our results underpin this advice and add that routine sessions may benefit from planning. We found that frequent, planned, bidirectional DO sessions in GP residency, initially and later on, are good practice, contributing to mimesis, feedback, assessment, entrustment, patient safety and a good training relationship. Supervisors can be stimulated to continue these sessions after the initial period.

Another shared recommendation from the literature^11^, ^26^, ^27^ is that the supervisor and resident openly discuss how DO can best help serve their mutual needs. In preparation for this dialogue, our results may inspire supervisors and residents to identify advantages and risks of the various patterns of DO.

### Implications for further research

Our findings raise several new questions that require further study. For instance, why do many supervisors cease planned DO sessions after the initiation phase? What would be the actual benefits and downsides of continued planned DO sessions in these training practices? An observational study of planned DO sessions, combined with interviews with residents and supervisors, could provide us with new insights here.

Because prolonged training relationships, such as exist in GP training, are not yet on the horizon in most PGME, it would be interesting to investigate whether planned bidirectional DO sessions could have similar benefits for shorter training relationships, both in the initial stage and later on.

Another interesting topic for further research is the patient's perspective on DO. How do patients experience the various aspects of the different patterns of DO and to what extent do they recognise the emotional discomfort that supervisors reported?

However, in anticipation of the limitations section, the first need is to complement our findings with a similar broad understanding of residents’ perspectives on DO.

### Limitations

We deliberately focused on a clearly defined phenomenon (i.e. observation of technical skills in one postgraduate GP programme). Yet, our findings shed new light on the role of observation in relation to the development of a trusting relationship between resident and supervisor that may transcend our research context.

We have, however, studied only supervisors’ perspectives. Residents may highlight other aspects of DO or look differently at the same issues; we found an example of the latter, comparing our study with that by Watling et al.,^15^ as indicated in the discussion.

Moreover, the specific context of GP residency has similarities, but also some distinct differences, with other postgraduate training programmes such as strong longitudinal training relationships and an extensive faculty development programme. Other professions and specialties have to judge to what extent our findings apply to their specific circumstances.

Our approach excluded DO of other than technical skills; the widespread use of video recording of consultations for the purpose of communication training in GP training programmes will probably generate different patterns of DO than we found by researching DO of technical skills.

Finally, a consequence of our research approach is that we have built our findings on what participants chose to share with us during the focus groups. We realise that some issues, such as falling short as a supervisor and the impact of competing demands on supervisors leading to less DO, might be informed by other research methods, such as observation in the context of ethnographic research.

## Conclusions

We have studied GP supervisors’ perspectives on the complexity of DO of technical skills. General practice supervisors balanced DO with nearby supervision and, in this process, DO meant much more than gathering information for purposes of feedback and assessment. We found four DO patterns that illustrate how DO helps to shape, and also reflects, the resident–supervisor relationship, and how DO can contribute to patient safety and residents’ learning. Tensions seemed to relate mostly to the ad hoc patterns of DO, whereas planned bidirectional DO sessions were considered a strategy that could prevent many of these tensions.

Our findings provide a more differentiated picture of DO in GP residency. Efforts to achieve more frequent DO for purposes of feedback and assessment have to take different patterns of DO into account. An open discussion between supervisors and residents on how DO can work best for both in different phases of the relationship seems a good starting point.

## Contributors

CBTR is the first researcher; he led all steps of the design of the study, data collection, coding, further analysis and interpretation of the data. He wrote all versions for revision and comments by the other authors and processed all comments until the final manuscript was reached. DH contributed substantially to the conception and design of the study; she organised the focus groups, transcribed most of the audiorecordings, helped analyse and code the transcripts and develop the code book and helped in further interpretation of the data. She revised the subsequent versions of the manuscript for important intellectual content. AHB contributed substantially to the conception and design of the study; she helped analyse and code the transcripts, helped develop the code book and helped in further interpretation of the data. She revised the subsequent versions of the manuscript for important intellectual content. HdV contributed substantially to the conception and design of the study; he helped code the transcripts and develop the code book; and helped in further interpretation of the data. He revised the subsequent versions of the manuscript for important intellectual content. FS contributed substantially to the conception and design of the study; he helped develop the code book, and helped in further interpretation of the data. He revised the subsequent versions of the manuscript for important intellectual content. AWMK contributed substantially to the conception and design of the study and helped with the interpretation of the data. She revised the subsequent versions of the manuscript for important intellectual content. PWT contributed substantially to the conception and design of the study; he helped code the transcripts and develop the code book, and helped in further interpretation of the data. He revised the subsequent versions of the manuscript for important intellectual content. All authors approved this final version for publication and have agreed to be accountable for all aspects of the work in ensuring that questions related to the accuracy or integrity of any part of the work are appropriately investigated and resolved.

## Funding

none.

##### Conflicts of interest

none.

##### Ethical approval

the study protocol was approved by the ethics review committee of the Dutch Association for Medical Education (Nederlandse Vereniging voor Medisch Onderwijs [NVMO]; NERB dossier no. 736).
